# Supplementary material for: Metal-organic framework patterns and membranes with heterogeneous pores for flow-assisted switchable separations
Source: Nat Commun. 2018 Sep 27;9:3968. doi: 10.1038/s41467-018-06438-0 (PMC6160406; doi:10.1038/s41467-018-06438-0)
Supplement: Supplementary file 1 — Supplementary Information [file 41467_2018_6438_MOESM1_ESM.pdf]

Supplementary Information

Metal-Organic Framework Patterns and Membrane with Heterogeneous Pores for Flow-assisted Switchable Separation

Guan-Young Jeong[1], Ajay K Singh[1], Min-Gyu Kim[2], Ki-Won Gyak[1], UnJin Ryu[3], Kyung Min Choi*[3] and Dong-Pyo Kim*[1]

[1]*Center of Intelligent Microprocess for Pharmaceutical Synthesis, Department of Chemical Engineering, POSTECH (Pohang University of Science and Technology), Pohang 790-784, Korea.*

[2]*Beamline Research Division, Pohang Accelerator Laboratory (PAL), POSTECH (Pohang University of Science and Technology), Pohang 376-73, Korea*

[3]*Department of Chemical and Biological Engineering, Sookmyung Women's University, 100 Cheongpa-ro 47 gil, Yongsan-gu, Seoul, 04310, Korea*

Corresponding Author

**E-mail: kmchoi@sm.ac.kr and dpkim@postech.ac.kr*

Supplementary Methods

Supplementary Materials

All chemicals were purchased from commercial sources and used without further purification: copper(II) nitrate ($Cu(NO_3)\cdot H_2O$, Sigma-Aldrich), trimesic acid (H_3BTC, Sigma-Aldrich), aluminum nitrate ($Al(NO_3)_3\cdot 9H_2O$, Sigma-Aldrich), terephthalic acid (H_2BDC, Sigma-Aldrich), europium(III) chloride ($EuCl_3$, Sigma-Aldrich), isophthalic acid (H_2IPA, Sigma-Aldrich), zirconium (IV) chloride ($ZrCl_4$, Sigma-Aldrich), 2,2′-Bipyridine-5,5′-dicarboxylic acid (H_2bpy, Sigma-Aldrich), iodomethane (ICH_3, Sigma-Aldrich), acetic acid (Sigma-Aldrich), potassium persulfate ($K_2S_2O_8$, Sigma-Aldrich) silver nitrate ($AgNO_3$, Sigma-Aldrich), N,N-dimethylformamide (DMF, Samchun chemicals), tetrahydrofuran (THF, Samchun chemicals), acetonitrile (ACN, Samchun chemicals), bovine serum albumin (lyophilized powder form, 66 KDa, Sigma-Aldrich), bovine hemoglobin (lyophilized powder form, 65 KDa, Sigma-Aldrich), fluorescein isothiocyanate-dextran (FITC-dextran, Sigma-Aldrich), anodized aluminum oxide (AAO, Whatman, 20 nm pore size, 25 mm diameter), Si wafer (P/100, dopant: boron, Semi-Materials Co., Ltd).

Supplementary Analysis and characterization

SEM images were collected using JEOL JSM-7401F high resolution scanning electron microscope operating at 3 kV. TEM images were collected using JEOL JEM-2200FS transmission electron microscope operating at 200 kV. UV-Vis absorption spectra were collected by a NanoDrop 2000c spectrophotometer (Thermo Fisher Scientific). The powder XRD diffractograms were obtained by a Rigaku X-ray diffractometer (Cu Kα radiation) at 100 kV, 30 mA. Gas adsorption isotherms were measured with a BELSORP-max automatic volumetric gas adsorption equipment. Typically, a sample of as-synthesized materials (~100

mg) was loaded and prior to the measurements, residual solvents was evacuated by heating at 100 ^{o}C under a high vacuum overnight. The zeta potentials of the N-quaternized MIL-53(Al)bpy-d40 membrane at different pH 4.7 and 7.0 were measured with a Zetasizer Nano MPT-2 (Malvern Instruments Ltd.). Fourier transform infrared (FT-IR) spectra were recorded in the range of 400-4000 cm^{-1} with FT/IR-4600 instrument equipped with a universal ZnSe ATR accessory. GC analysis was performed on an Agilent Technologies 7890A gas chromatography (Agilent Tech., USA/Germany) using fused silica capillary column (column, 0325mm x 30m).

Ex-situ X-ray absorption spectroscopy

For X-ray absorption spectra, Ag K-edge X-ray absorption near edge structure (XANES), Cu K-edge XANES and Cu K-edge extended X-ray absorption fine structure (EXAFS) spectra were collected on BL10C beam line at the Pohang light source (PLS-II) with top-up mode operation under a ring current of 200 mA at 3.0 GeV. The monochromatic X-ray beam could be obtained using liquid-nitrogen cooled Si (111) double crystal monochromator (Bruker ASC) with in-situ exchange under the vacuum from high intense X-ray photons of multipole wiggler source. The Si (111) crystal pair has been used for each atom K-edge XAFS measurement. The X-ray absorption spectroscopic data were recorded for the uniformly dispersed powder samples, freshly taken from the reaction vessel, with a proper thickness on the polyimide film, in transmission mode with N_2 gas-filled ionization chambers as photon detectors. Higher order harmonic contaminations were eliminated by detuning to reduce the incident X-ray intensity by ~40%.

Supplementary Notes

Supplementary Preparation of MOF particles.

HKUST-1: HKUST-1 was followed by reported method[1]. HKUST-1 precursor solution was prepared by adding $Cu(NO_3)·H_2O$ (62 mg, 0.33 mmol) and H_3BTC (70 mg, 0.33 mmol) to homogeneous solution of 5 mL of *N*,*N*-dimethylformamide (DMF), 10 mL of ethanol (EtOH) and 10 mL of deionized water (H_2O). This mixture was stirred for 1 hr until complete dissolution of the metallic salt and the organic ligand, then transferred into 40 mL Teflon-lined autoclave. It was kept at 85 ^oC for 20 hrs in oven to yield small crystals. Teflon-lined autoclave was cooled down to room temperature naturally and the synthesized products were collected by centrifuging (4000 rpm, 15 min). Obtained products were washed several times with fresh EtOH, the resultant products were dried at 60 ^oC overnight under vacuum.

MIL-100(Al): MIL-100(Al) synthesis was followed by reported method[2]. Typically, MIL-100(Al) precursor solution was prepared by adding $Al(NO_3)_3·9H_2O$ (70 mg, 0.33 mmol) and H_3BTC (70 mg, 0.33 mmol) into 20 mL of H_2O. The precursor was sonicated for 30 min till clear and transparent solution, the precursor was transferred into Teflon-lined autoclave and placed in a preheated oven at 220 ^oC for 1 day. After cooling to room temperature, the resulted products were collected by centrifugation (4200 rpm, 12 min), and washed several times with EtOH and deionized water, and dried at 60 ^oC overnight under vacuum.

EuMOF: EuMOF synthesis was followed by reported method[3]. Typically, EuMOF precursor solution was prepared by mixing of $Eu(NO_3)_3•5H_2O$ (86 mg, 0.33 mmol) and H_2IPA (55 mg, 0.33 mmol) into 25 mL DMF. The precursor was sonicated for 30 min, the precursor was transferred into Teflon-lined autoclave and placed in a preheated oven at 140 ^oC for 12 hrs. After cooling to room temperature, the resulted products were collected by centrifugation (4200 rpm, 12 min), and washed several times with EtOH, and dried at 80 °C overnight under vacuum.

UiO-66: UiO-66 synthesis was followed by reported method[4]. Typically, $ZrCl_4$ (0.7 g, 3 mmol) and H_2BDC (0.5 g, 3 mmol) were dissolved in 180 mL DMF *via* ultrasonication for 30 min. 25 mL of acetic acid was added into this mixture. The mixture was then sealed in a 30 mL Teflon reactor and maintained at 90 ^oC for 1 day. After the reaction being cooled down to room temperature, the precipitate was recovered by centrifugation and washing with DMF for several times. Then, the solids were suspended in ethanol for activation. Finally, the resulting white powder was dried in vacuum oven at 60 ^oC for overnight.

MIL-53(Al): MIL-53(Al) synthesis was followed by reported method[5]. Typically, MIL-53(Al) precursor was prepared by mixing of $Al(NO_3)_2·6H_2O$ (70 mg, 0.33 mmol) and H_2BDC (55 mg, 0.33 mmol) into 20 mL of H_2O. The precursor was sonicated for 30 min, the precursor was transferred into Teflon-lined autoclave and placed in a preheated oven at 220 ^oC for 3 days. After cooling to room temperature, the resulted products were separated by centrifugation (4200 rpm, 12 min), and washed several times with EtOH and deionized water mixture, and dried at 80 °C overnight under vacuum.

MIL-101(Cr): MIL-101(Cr) synthesis was followed by reported method[6]. Typically, MIL-101(Cr) precursor was prepared by mixing of $Cr(NO_3)_3·9H_2O$ (400 mg, 1 mmol), H_2BDC (164 mg, 1 mmol), HF (1 mmol) into 4.8 ml H_2O. The mixture was sonicated for 30 min, the precursor was transferred into Teflon-lined autoclave and placed in a preheated oven at 220 ^oC for 8 hrs. After cooling naturally to room temperature, the resulted products were separated by centrifugation (4200 rpm, 12 min) and washed several times with DMF, EtOH and deionized water, and finally dried at 80 ^oC overnight under vacuum.

MIL-100(Fe): MIL-100(Fe) synthesis was followed by reported method[2]. Typically, MIL-100(Fe) precursor was prepared by mixing of $FeCl_3·6H_2O$ (648 mg), H_3BTC (552 mg), HF

(0.16 ml) and HNO_3 (0.252 ml) into 20 ml H_2O. The mixture was sonicated for 30 min, the precursor was transferred into Teflon-lined autoclave and placed in a preheated oven at 150 ^oC for 6 days. After cooling naturally to room temperature, the resulted products were separated by centrifugation (4200 rpm, 12 min) and washed several times with DMF, EtOH and deionized water, and finally dried at 80 ^oC overnight under vacuum.

Supplementary Preparation of UiO-66-d patterns

Pre-patterning on Si-wafer or glass slide: Si wafer or glass slide was carefully cleaned with piranha solution at 70 ^oC for 40 min, followed fresh EtOH and acetone, dried with blowing N_2 and entirely plasma treated for 1 min. 5 mL of thioglycolic acid was introduced into 20 mL EtOH, then sonicated for 10 min. This initiator solution was taken by pipette with 5-20 µL tip. Later, tip was gently touched on substrate and slowly followed with desired location. This initiator/substrate was kept on heater at 60 ^oC for 2 hrs, finally pre-patterned substrate was obtained.

Pre-patterning on polyimide: Polydopamine solution was synthesized by previously reported method[7]. Polyimide film was horizontally immersed into dopamine hydrochloride solution in 10 mM Tris-HCL buffer for 12 hrs. After coating, the substrate was washed with deionized water and EtOH, then dried in a vacuum oven for overnight.

Growth of UiO-66 pattern on various substrates: UiO-66 precursors were prepared by mixing $ZrCl_4$ (77 mg, 0.33 mmol) and H_2BDC (55 mg, 0.33 mmol) into the 20 mL of DMF. In case of pre-patterned Si wafer or glass slide, substrate and UiO-66 pattern precursor were introduced into the 100 mL autoclave. The crystallization of UiO-66 on pre-patterned Si wafer or glass slide was conducted at 80 ^oC for 1 hr. In case of pre-patterned polyimide, UiO-66 precursor was transferred on pre-patterned polyimide with desired location by pipette with 5-20 µL tip, then gently moved on heater. The crystallization of UiO-66 on pre-patterned

polyimide was conducted at 80 ^{o}C for 1 hr. After cooling to room temperature, the UiO-66 pattern was gently washed several times with EtOH and deionized water mixture, and dried at 80 °C overnight under vacuum.

Decarboxylation for UiO-66-d40 patterns: The etching solution was prepared by mixing 25 mg of $AgNO_3$, 25 mg of $K_2S_2O_8$ in the 20 ml of acetonitrile (ACN), then sonicated for 30 min. The UiO-66 patterns on various substrates were horizontally immersed into etching solution, the mixture was transferred into 50 mL of Teflon-lined autoclave, and placed in a preheated oven. The decarboxylation of UiO-66 patterns was conducted at 150 ^{o}C for 40 min. When the reaction was finished, autoclave reactor was quickly moved into ice bath to quench and prevent further etching. After cooled down to room temperature, the substrate was rinsed with deionized water for three times. The UiO-66-d patterns was obtained and finally dried in the vacuum oven at 70 ^{o}C for overnight.

Supplementary Preparation of MIL-53(Al)-d40 membrane

Water flux measurement: The water flux was measured in a water permeation experiment through the MOF membrane (25 mm diameter) combined AAO holder with assumption of pressure of 1 bar.

Growth of MIL-53(Al) on AAO membrane: Our experiment was simple conversion as AAO + H_2BDC -> MIL-53 on AAO. First, a round AAO disc with 25 mm of diameter and 20 nm pore size was washed with deionized water, and was placed at the bottom of the Teflon-lined autoclave (25 ml). Then, mixture of H_2BDC (0.166 g, 1 mmol) and H_2O (10 ml) was gently added in the autoclave. After sealing, the autoclave was placed in a preheated oven at 140 ^{o}C for 1-4 hrs. Naturally cooling to room temperature, the resulted disc was carefully collected and washed with EtOH several times and dried in 70 ^{o}C vacuum oven for overnight.

Growth of MIL-53(Al)bpy on AAO membrane: All conditions are exactly the same as MIL-53(AL) membrane, except replacing H_2BDC by 2,2'-bipyridine-5,5'-dicarboxylic acid (bpy-H_2BDC) to give the pyridine sites into MIL-53(Al).

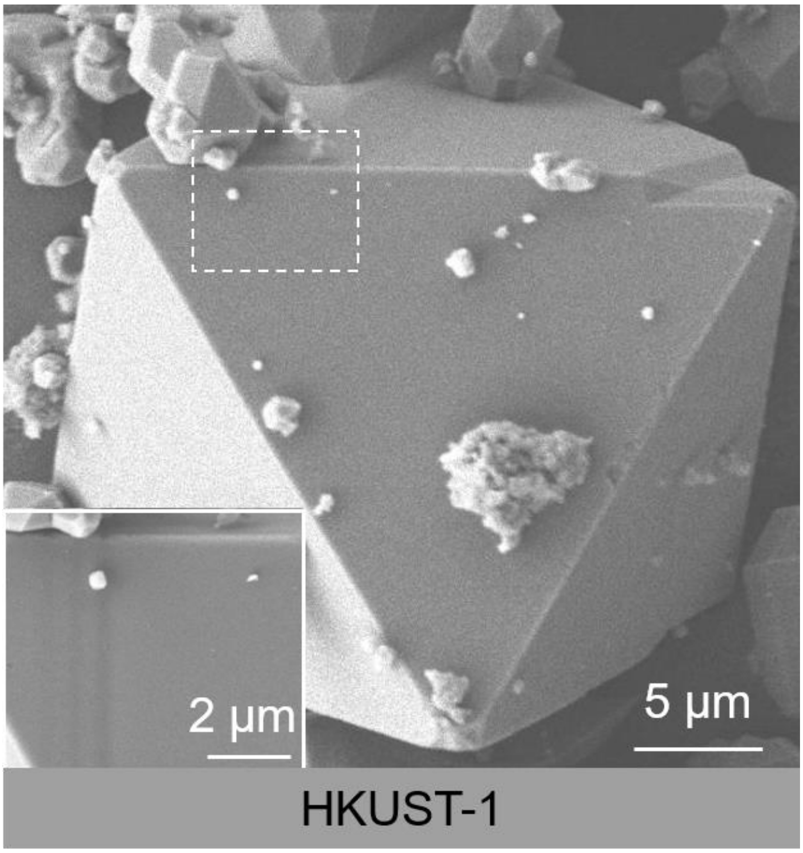

Supplementary Figure 1. SEM image of the pristine HKUST-1. The left bottom inset is the high magnification image of the dotted line part.

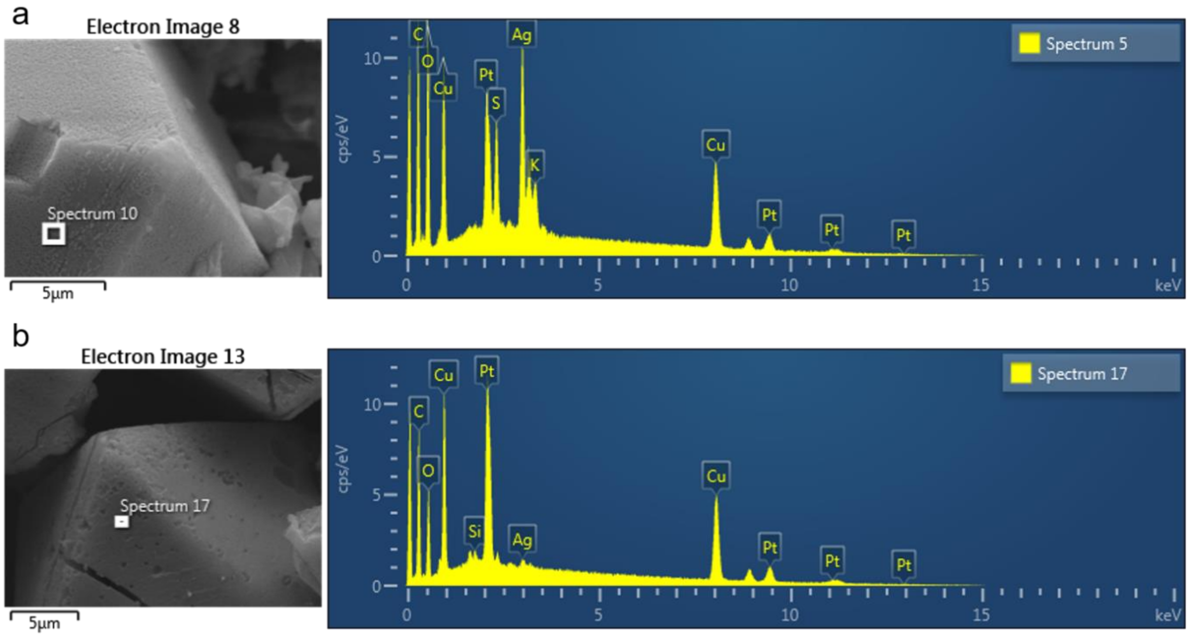

Supplementary Figure 2. Energy-dispersive X-ray spectroscopy (EDX) of HKUST-1-d40 samples (a) before and (b) after washing steps. The decarboxylated samples were washed thoroughly three times with fresh DI-water and finally one time with EtOH to remove all adsorbed Ag and K ions.

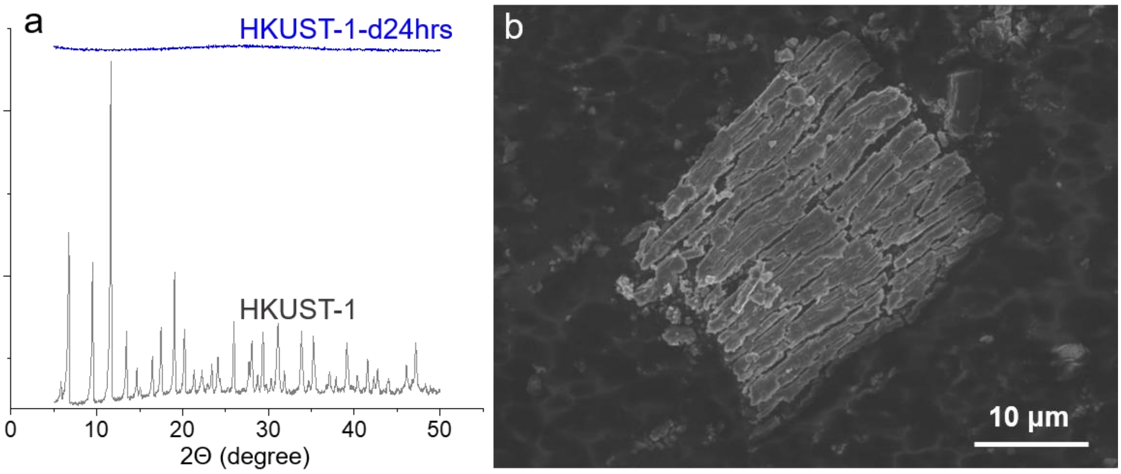

Supplementary Figure 3. (a) X-ray diffraction of HKUST-1 and the decarboxylated for 24 hrs (HKUST-1-d24hrs), (b) SEM images of HKUST-1-d24hrs.

Supplementary Table 1. Summarized pore analysis data of decarboxylated HKUST-1 at 120 ^{o}C for various times.

	$^{a}S_{BET}$ (m^2g^{-1})	$^{b}V_t$ (cm^3g^{-1})	$^{c}V_{micro}$ (cm^3g^{-1})	$^{d}V_{meso}$ (cm^3g^{-1})	^{e}gain V_{meso}/loss V_{micro}
HKUST-1	1690	0.3637	0.3637	0	
HKUST-d10	1581	0.4995	0.351	0.1485	11.69
HKUST-d20	1638	0.5706	0.3105	0.2601	4.89
HKUST-d30	1326	0.5748	0.3074	0.2674	4.75
HKUST-d40	1308	0.6833	0.2526	0.4307	3.88
HKUST-d60	1191	0.7351	0.2114	0.5237	3.44
HKUST-d90	874	0.8157	0.1875	0.6282	3.57

$^{a}S_{BET}$: BET specific surface area, $^{b}V_t$: total specific pore volume, $^{c}V_{micro}$: specific micropore volume calculated by HK method, $^{d}V_{meso}$: specific mesopore volume calculated by BJH analysis, ^{e}Calculated ratio by V_{meso}/(V_t of HKUST-1 − V_{micro} of HKUST-dx)

Supplementary Table 2. Summarized pore analysis data from the reported literatures[i].

Etching method	MOF species	[a]S_{BET} (m^2g^{-1})	[b]V_t (cm^3g^{-1})	[c]V_{micro} (cm^3g^{-1})	[d]V_{meso} (cm^3g^{-1})	[e]gain V_{meso}/ loss V_{micro}	Ref.
Water	[f]POST-66(Y)	2400	0.820	0.820	0	1.80	8
		1580	1.023	0.568	0.455		
H_3PO_4	[g]MIL-100(Fe)	2020	0.8918	0.8561	0.1059	1.11	9
		730	0.7113	0.4161	0.5986		
HCl	[h]UiO-66	1204	0.59	0.59	0	1.18	10
		1018	0.65	0.27	0.38		

[a]S_{BET}: BET specific surface area, [b]V_t: total specific pore volume, [c]V_{micro}: specific micropore volume calculated by HK method, [d]V_{meso}: specific mesopore volume calculated by BJH analysis, [e]Calculated ratio by V_{meso}/(V_t of HKUST-1 − V_{micro} of HKUST-dx). [f]POST-66(Y): etching was conducted by immersing in the water at RT for 10 min. [g]MIL-100(Fe): etching was treated by immersing in the 6 ml of 20 mM H_3PO_4 solution for 5 hrs. [h]UiO-66: nano-sized and acid-sensitive MOF-5 was used as template, etching was conducted by dispersing the UiO-66 in the 10 ml of diluted HCl solution (pH=1) for 10 min. [i]Every upper row was about pristine MOFs information, while every lower row was about MOFs information after etching.

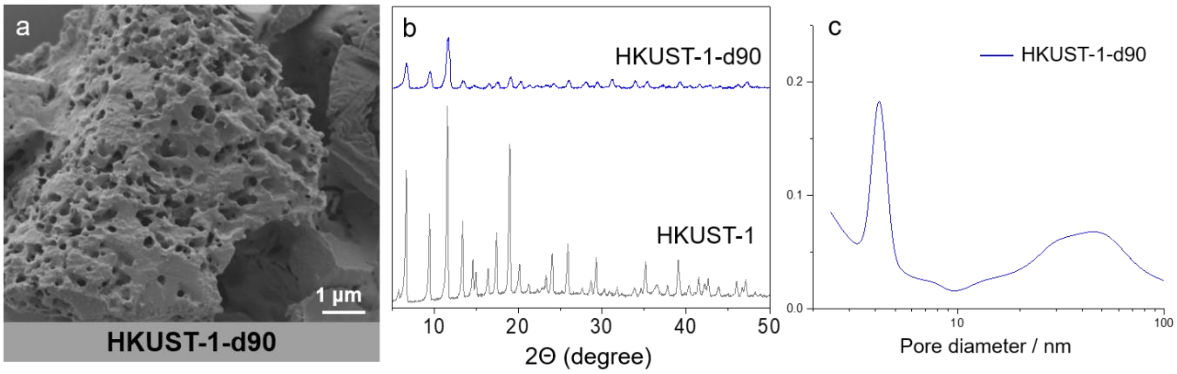

Supplementary Figure 4. (a) SEM image, (b) X-ray diffraction and (c) pore size distribution of HKUST-1-d90 at 120 ºC.

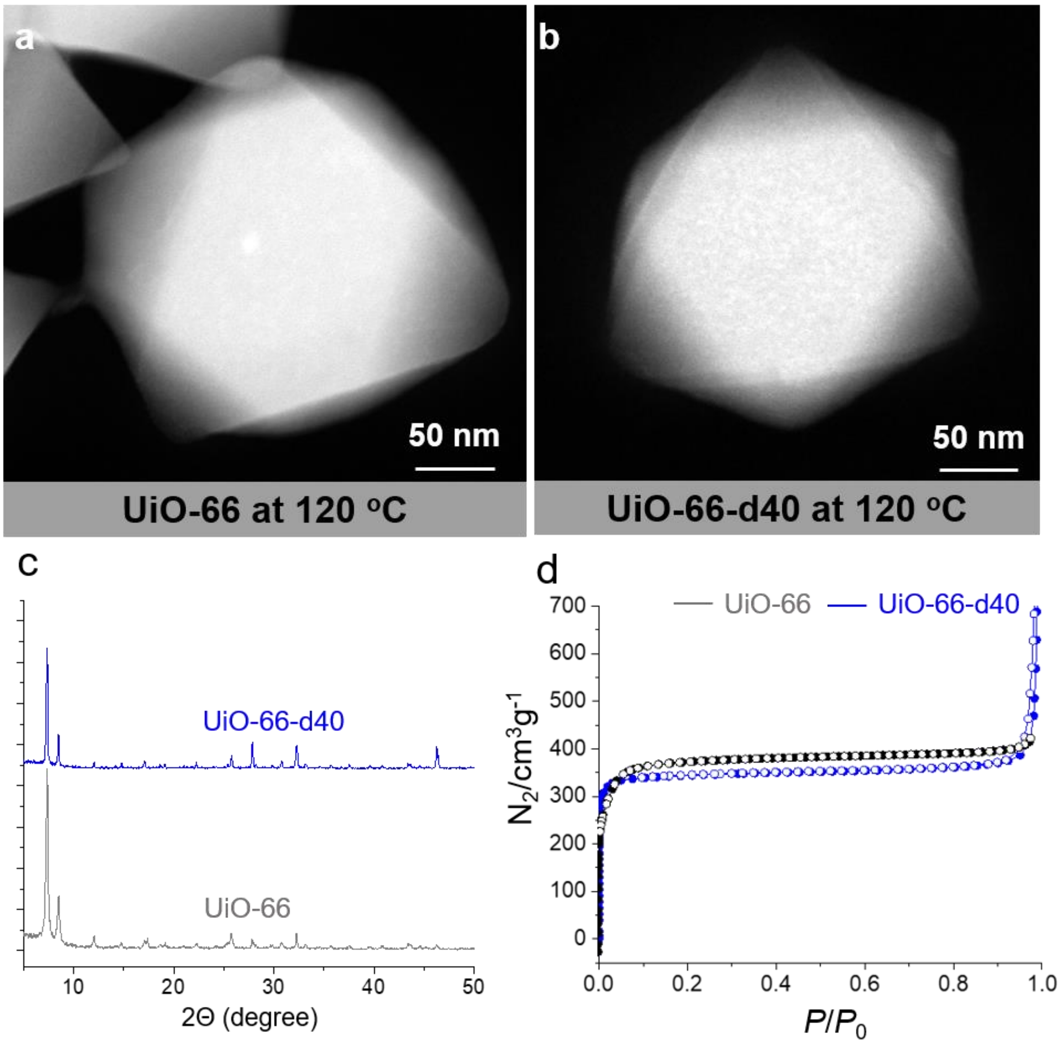

Supplementary Figure 5. Decarboxylation of UiO-66 into UiO-66-d40 at 120 °C for 40 min. TEM images of (a) UiO-66, (b) UiO-66-d40 at 120 °C reaction temperature, (c) X-ray diffraction and (d) N_2 adsorption.

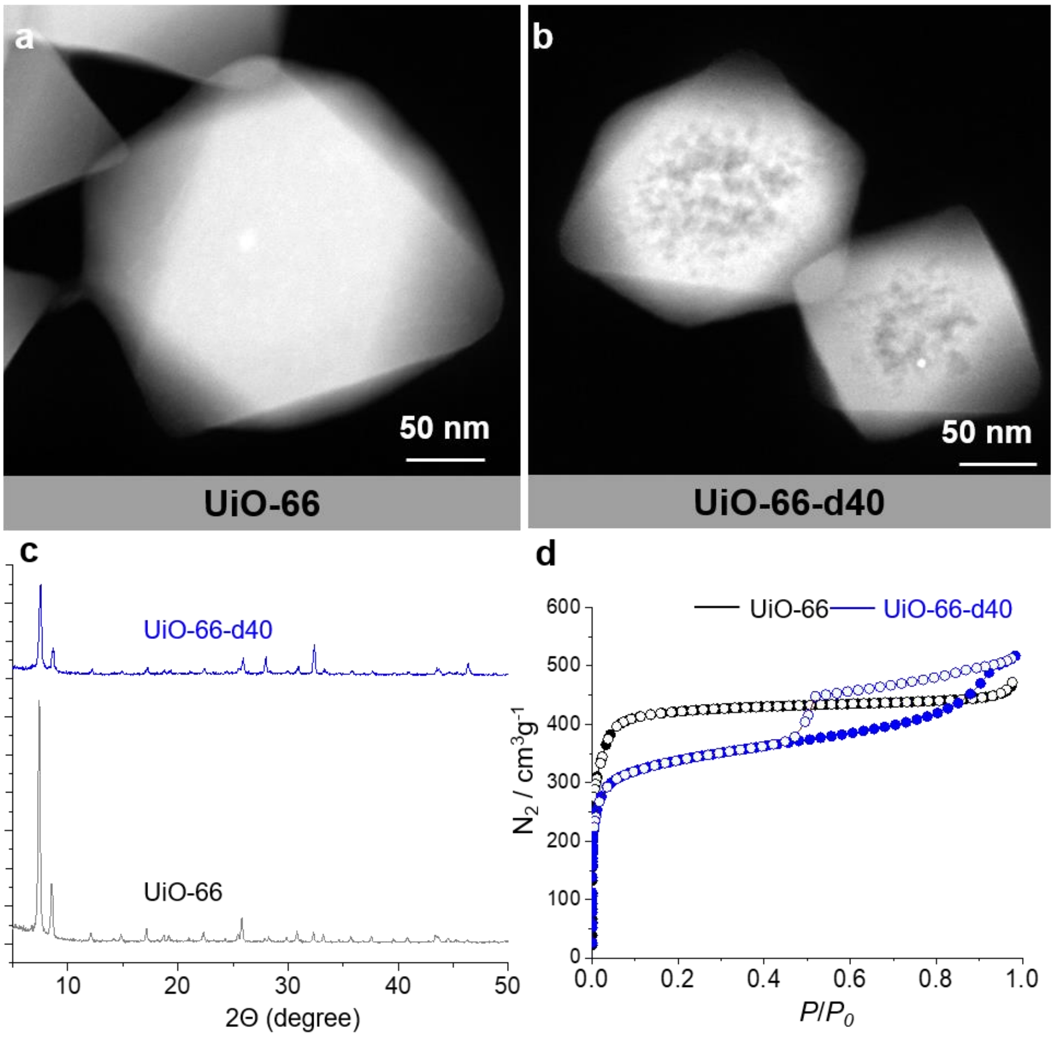

Supplementary Figure 6. Transformation to hierarchical micro- and meso-porous structures in a microporous UiO-66 by decarboxylation at 150 ºC for 40 min. TEM images of (a) UiO-66, (b) UiO-66-d40, (c) X-ray diffraction and (d) N_2 sorption.

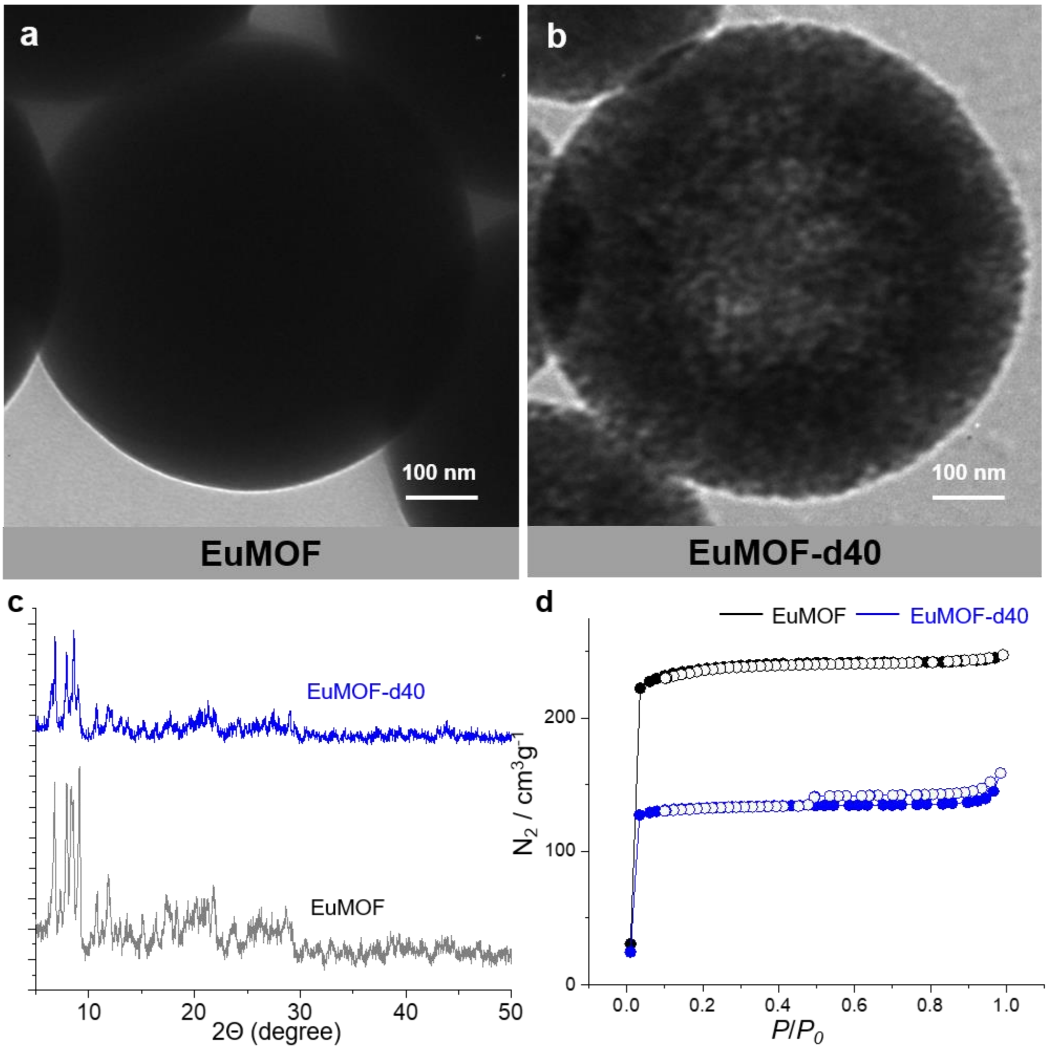

Supplementary Figure 7. Transformation to hierarchical mesoporous structures in a microporous EuMOF by decarboxylation at 150 ºC for 40 min. TEM images of (a) EuMOF, (b) EuMOF-d40, (c) X-ray diffraction and (d) N_2 sorption.

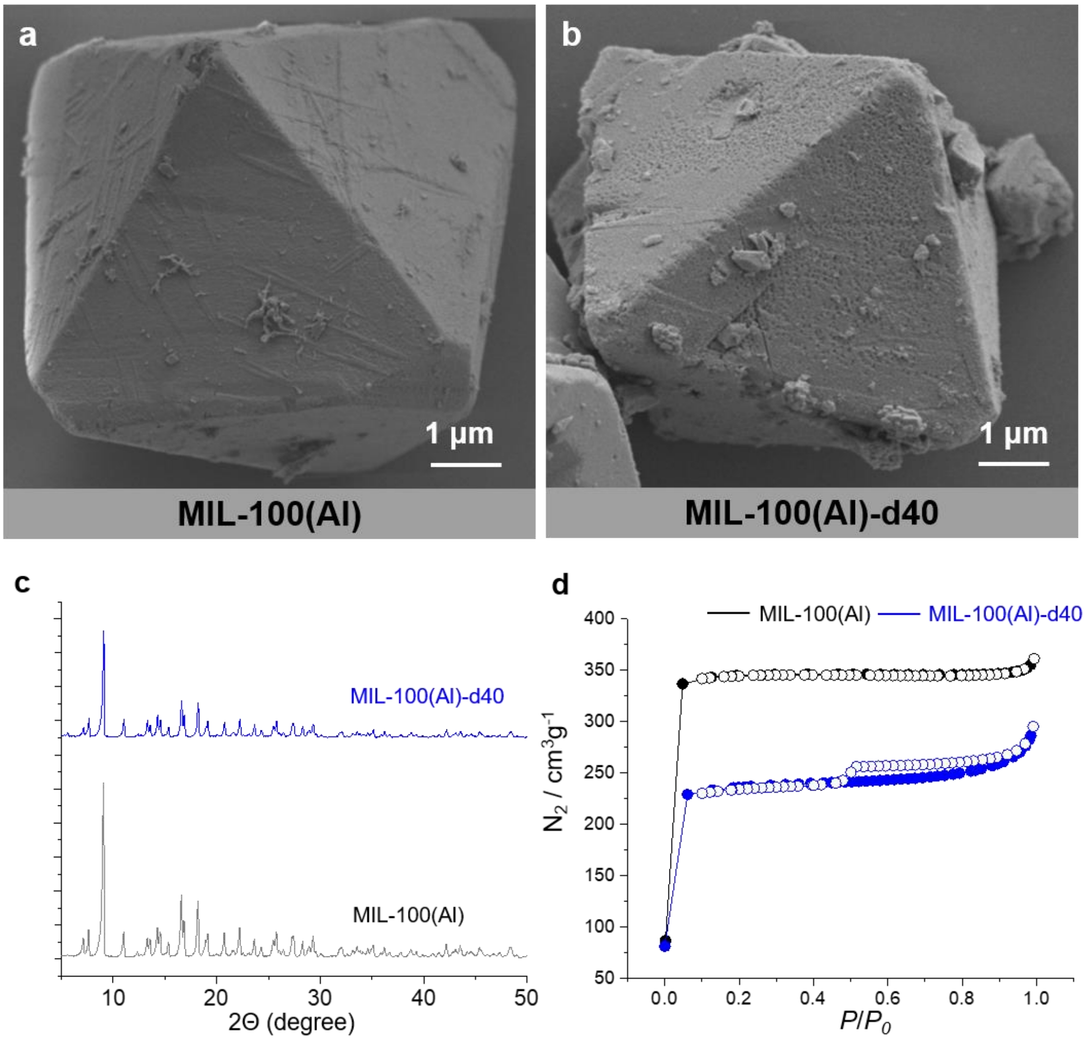

Supplementary Figure 8. Transformation to hierarchical mesoporous structures in a microporous MIL-100(Al) by decarboxylation at 150 ºC for 40 min. SEM images of (a) MIL-100(Al), (b) MIL-100(Al)-d40, (c) X-ray diffraction and (d) N_2 sorption.

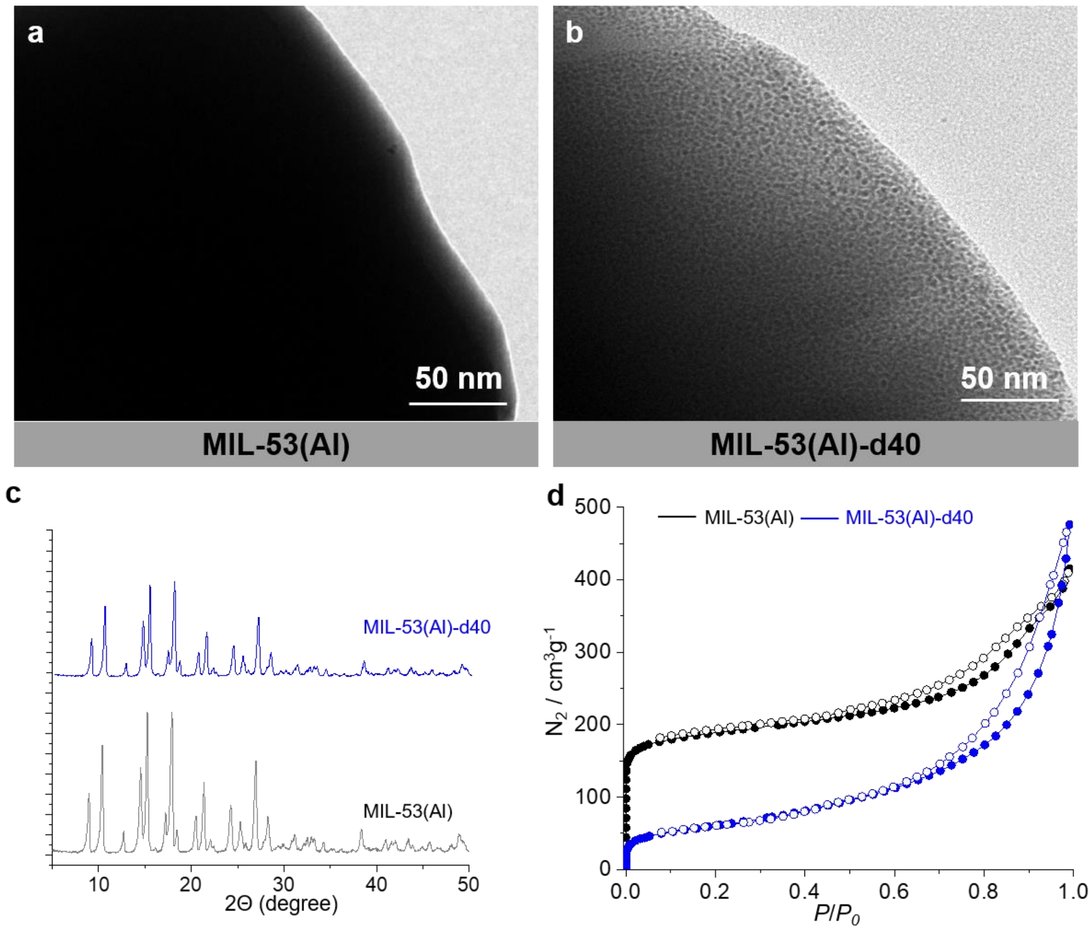

Supplementary Figure 9. Transformation to hierarchical mesoporous structures in a microporous MIL-53(Al) by decarboxylation at 150 ºC for 40 min. TEM images of (a) MIL-53(Al), (b) MIL-53(Al)-d40, (c) X-ray diffraction and (d) N_2 sorption. The larger pores in MIL-53(Al) is presumably originated from the inter-particle space.

Supplementary Table 3. Porosity analysis data of MIL-100 (Al), EuMOF and MOF-5 by decarboxylation etching chemistry at 150 °C.

	$^{a}S_{BET}$ (m^2g^{-1})	$^{b}V_t$ (cm^3g^{-1})	$^{c}V_{micro}$ (cm^3g^{-1})	$^{d}V_{meso}$ (cm^3g^{-1})	^{e}gain V_{meso}/ loss V_{micro}
UiO-66	1720	0.7702	0.6696	0.1006[f]	4.97
UiO-66-d40	1226	1.4091	0.5088	0.9003	
EuMOF	620	0.1654	0.1654	0	2.30.
EuMOF-d40	326	0.2545	0.1074	0.1471	
MIL-100(Al)	1620	0.4196	0.4196	0	3.20
MIL-100(Al)-d40	1214	0.7234	0.2813	0.4421	
MIL-53(Al)[g]	722	0.6385	0.6110	0.0275[f]	1.36
MIL-53(Al)-d40[g]	218	0.7214	0.3773	0.3441	

$^{a}S_{BET}$ is the BET specific surface area. $^{b}V_t$ is total specific pore volume. $^{c}V_{micro}$ is the specific micropore volume calculated by HK method. $^{d}V_{meso}$ is the specific mesopore volume calculated by BJH analysis. ^{e}Calculated ratio by V_{meso}/(V_t of the pristine − V_{micro} of the decarboxylated). $^{f}V_{meso}$ for the pristine UiO-66 and MIL-53(Al) is originated from the inter-particle space as their particle sizes is small. ^{g}Pretreatment of MIL-53(Al) and MIL-53(Al)-d40 at 400 °C might affect the original pore characteristics.

The surface area of the decarboxylated EuMOF at 150 °C for 40 min decreased from 620 m^2/g to 326 m^2/g while the total pore volume increased from 0.16 cm^3/g to 0.25 cm^3/g. The micropore volume of EuMOF-d40 was preserved as 62% of the pristine EuMOF and the ratio of mesopore gain/micropore loss is 2.30. The surface area of pristine MIL-100 (Al) (1620 m^2/g) was decreased to 1214 m^2/g while total pore volume was increased from initial 0.41 cm^3/g to 0.72 cm^3/g. The micropore volume of MIL-100-d40 was remained as 68% of the pristine MIL-100 and the ratio of mesopore gain/micropore loss is 3.20. The surface area of the decarboxylated MIL-53(Al) decreased from 722 m^2/g to 218 m^2/g while the total pore volume increased from 0.64 cm^3/g to 0.72 cm^3/g. The micropore volume of MIL-53(Al) was preserved at 62% of the pristine MIL-53(Al) and the lower ratio, 1.36, of mesopore gain to micropore loss was presumably due to higher desorption pretreatment at 400 °C that might collapse the micropores.

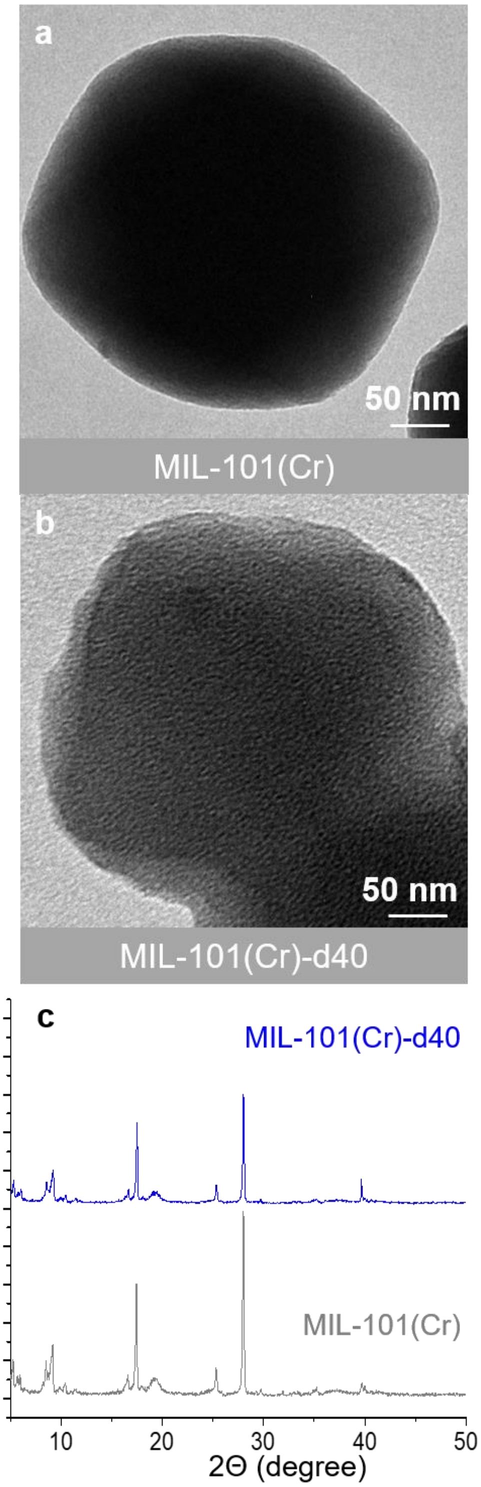

Supplementary Figure 10. Transformation to hierarchical mesoporous structures in a microporous MIL-101(Cr) by decarboxylation at 150 ^{o}C for 40 min. TEM images of (a) MIL-101(Cr), (b) MIL-101(Cr)-d40, (c) X-ray diffraction.

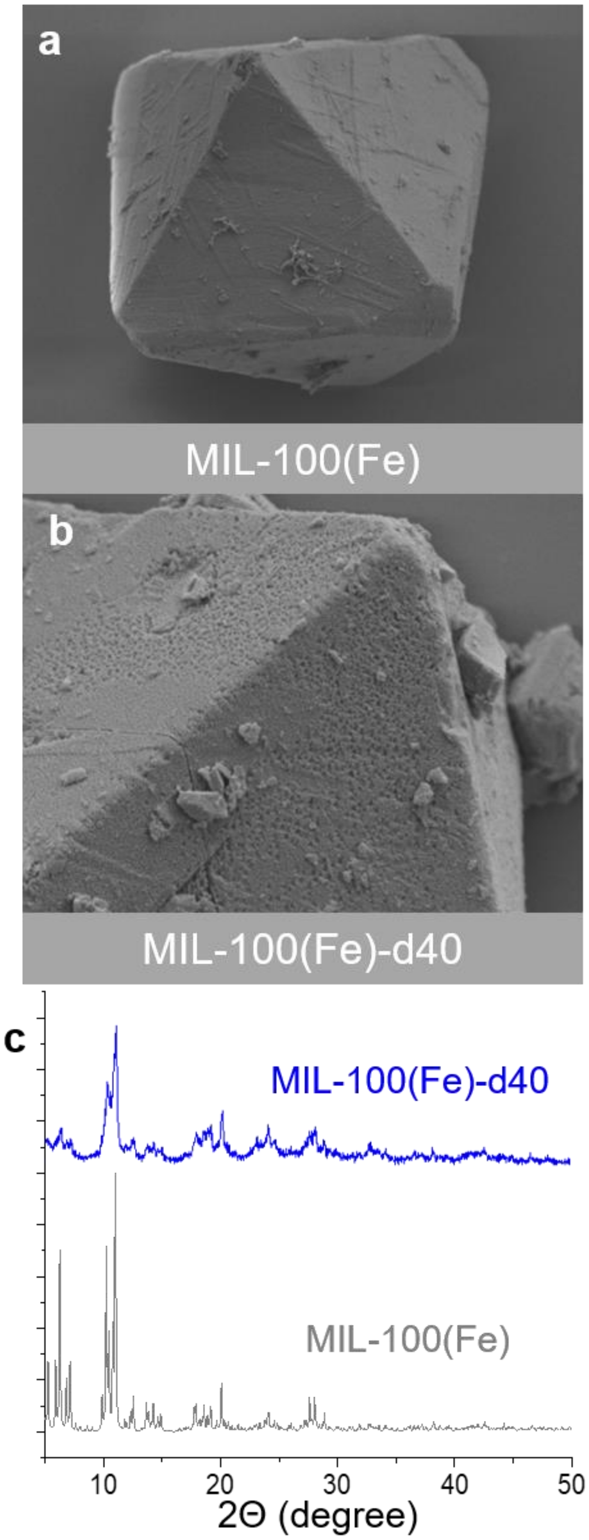

Supplementary Figure 11. Transformation to hierarchical mesoporous structures in a microporous MIL-100(Fe) by decarboxylation at 150 ºC for 40 min. TEM images of (a) MIL-100(Fe), (b) MIL-100(Fe)-d40, (c) X-ray diffraction.

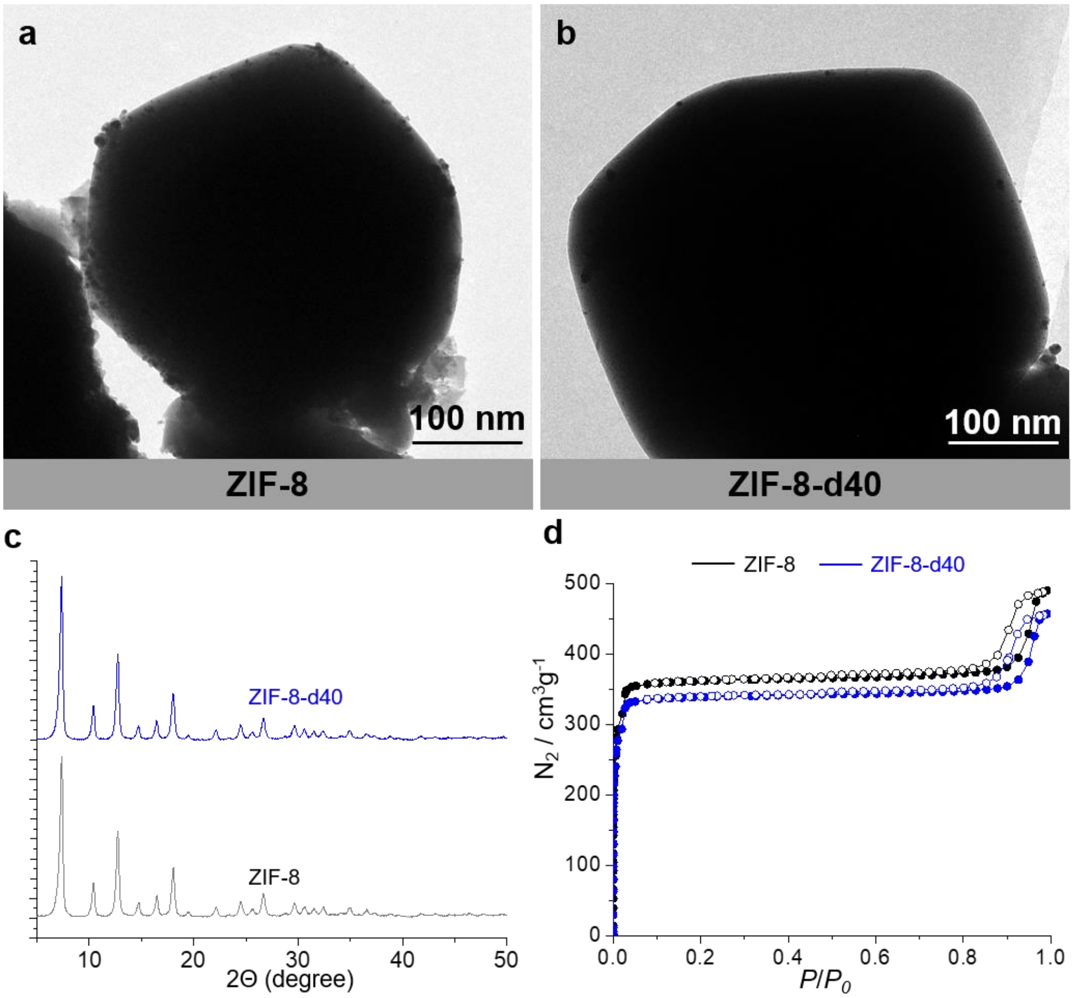

Supplementary Figure 12. TEM images of (a) ZIF-8, (b) decarboxylated ZIF-8-d40 at 150 °C for 40 min, (c) X-ray diffraction and (d) N_2 sorption.

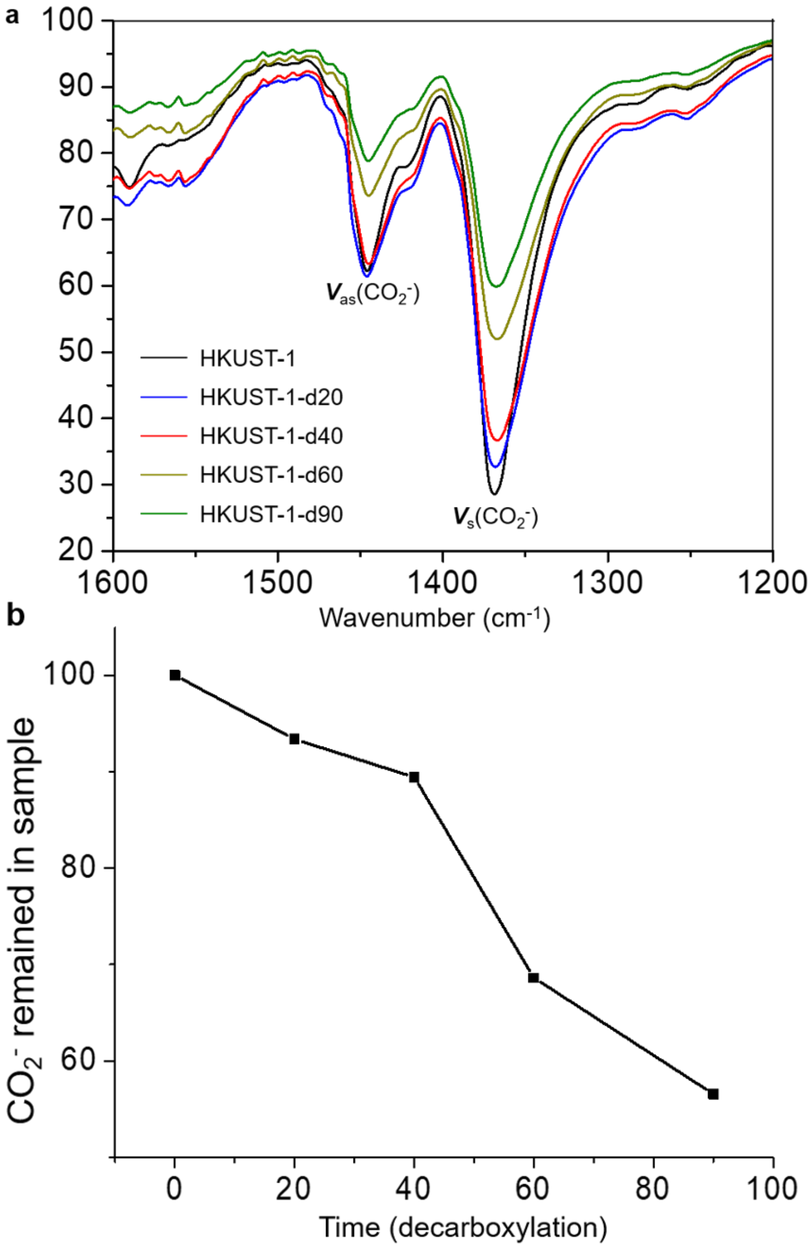

Supplementary Figure 13. (a) ATR-IR spectroscopy of HKUST-1-d samples and the normalization of the integrated carboxyl peak intensities.

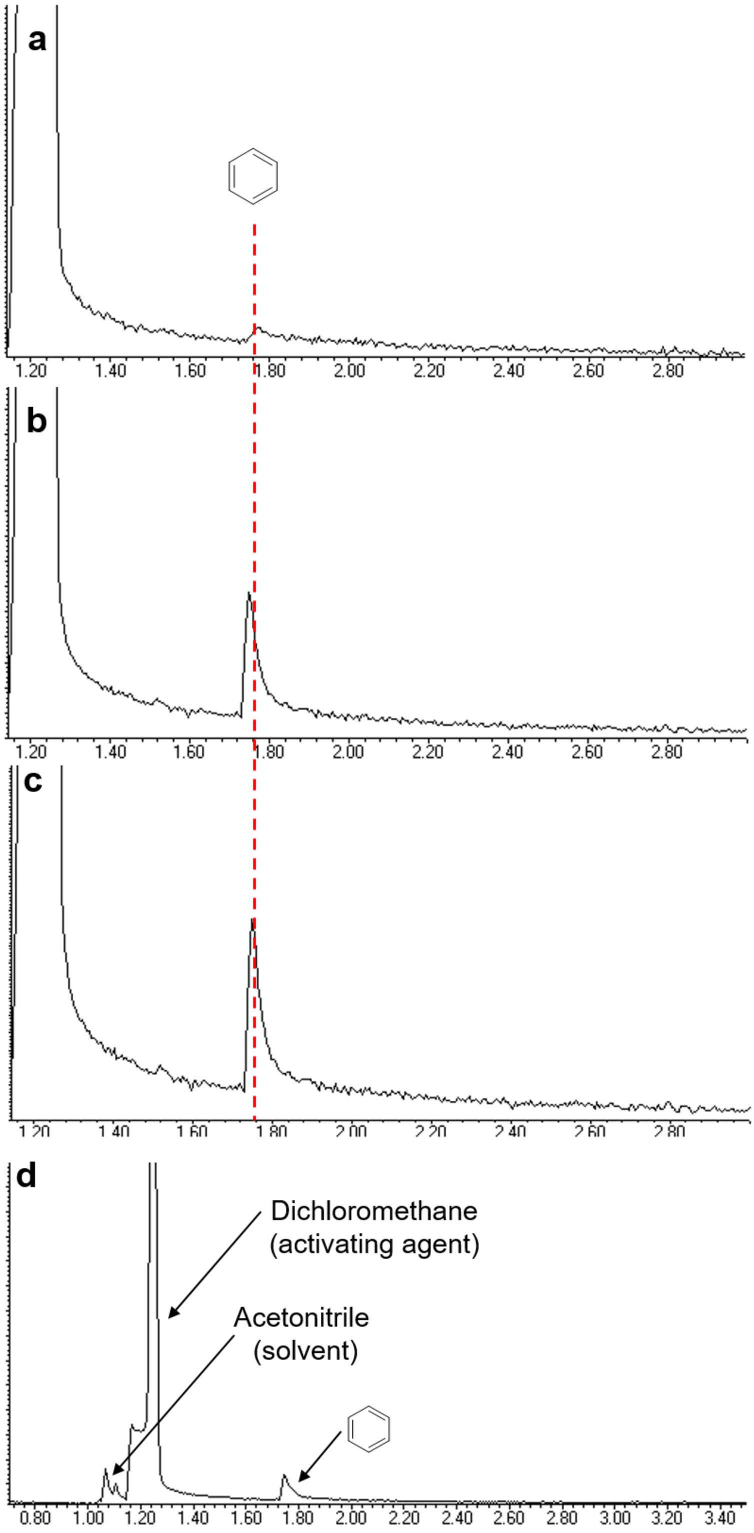

Supplementary Figure 14. GC-MS spectra of supernatant etching solutions obtained from (a) HKUST-1-d20, (b) -d40 and (c) -d60 samples from 1.1-3 min running time, (d) reduced GC-MS spectra of HKUST-1-d60 sample from 0.75-3.5 min running time. Note that the detected dichloromethane (DCM) was originated to the activating additive for solvent switching during synthesis of HKUST-1, which was weakly coordinated to the metal.

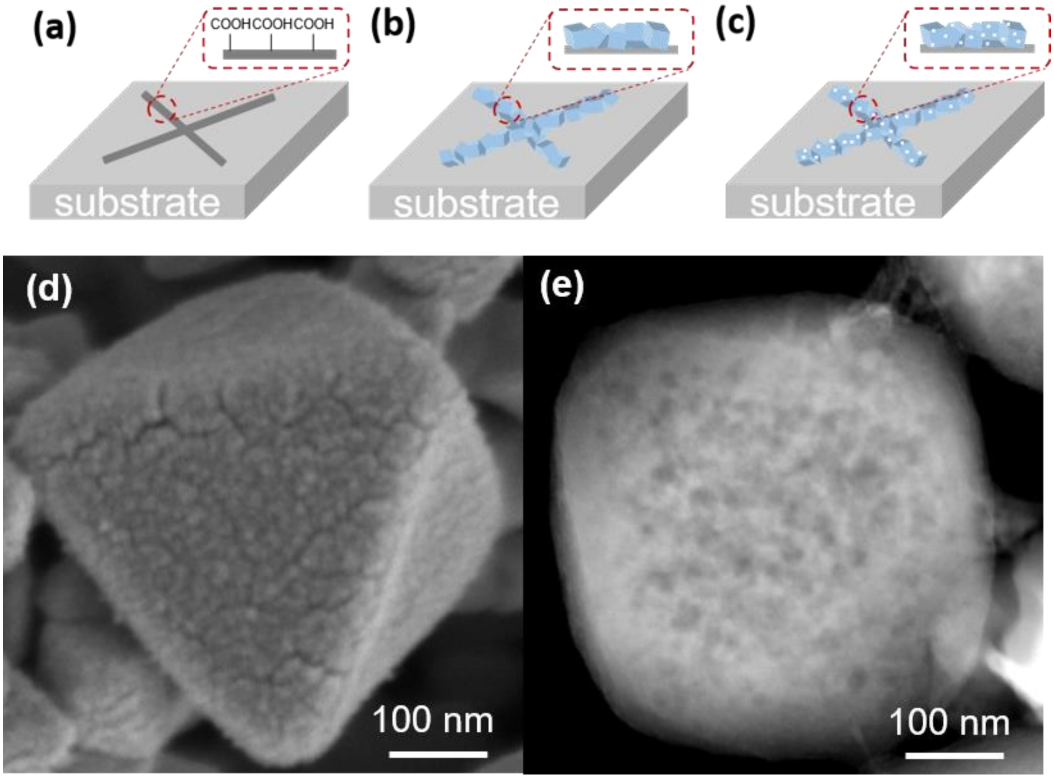

Supplementary Figure 15. (a-c) Conceptual illustration for the sequential process of UiO-66-d40 pattern formation via a serial surface modification, MOF growth, and decarboxylation. (d and e) SEM and TEM images of the UiO-66-d40 particles removed from Si substrate.

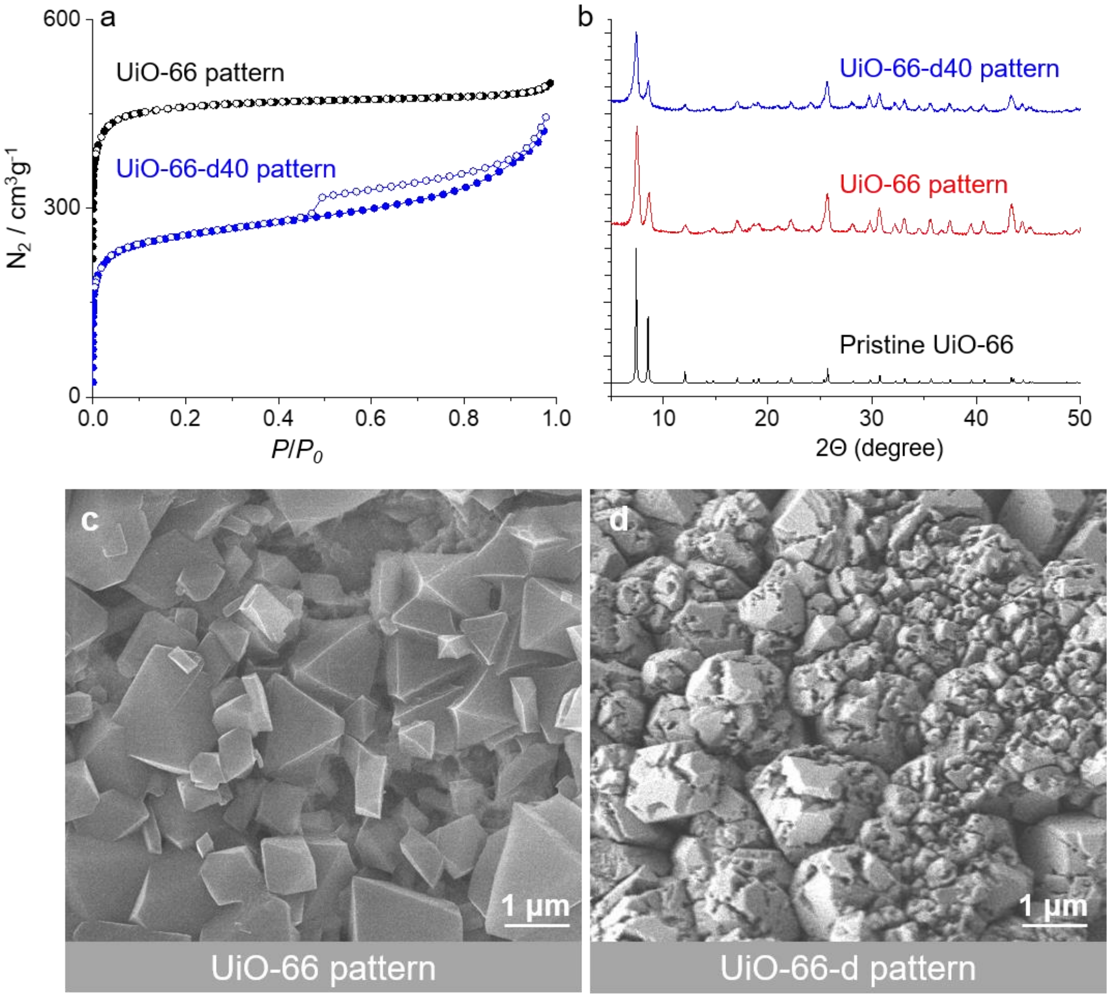

Supplementary Figure 16. (a) N_2 sorption, (b) X-ray diffraction, SEM images of (c) UiO-66 pattern and (d) decarboxylated UiO-66-d40 pattern at 150 °C for 40 min, then collected by detaching from glass substrate.

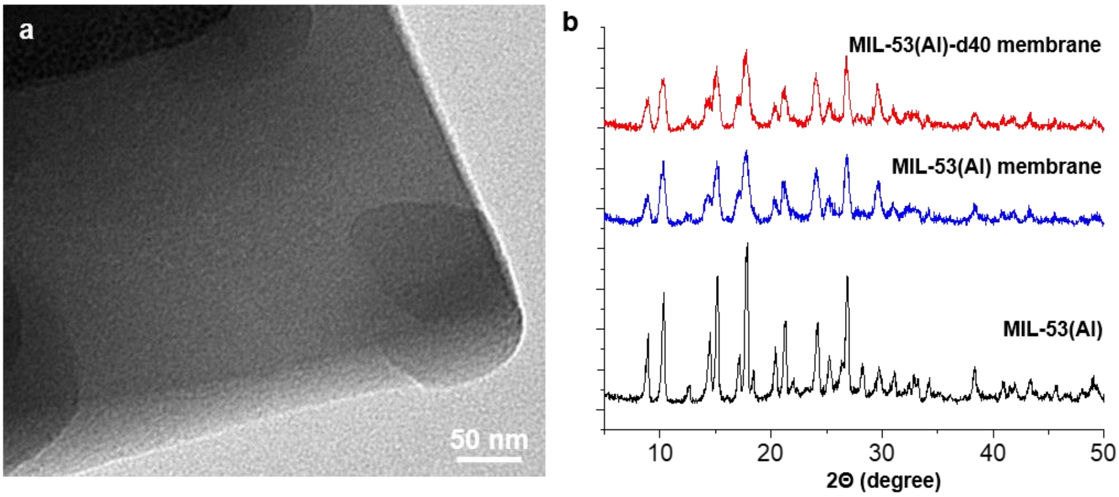

Supplementary Figure 17. (a) TEM image of the detached sample from decarboxylated MIL-53(Al)-d40 membrane at 150 ºC for 40 min and (b) X-ray diffraction patterns of MIL-53(Al), the detached sample from MIL-53(Al) membrane and MIL-53(Al)-d40 membrane.

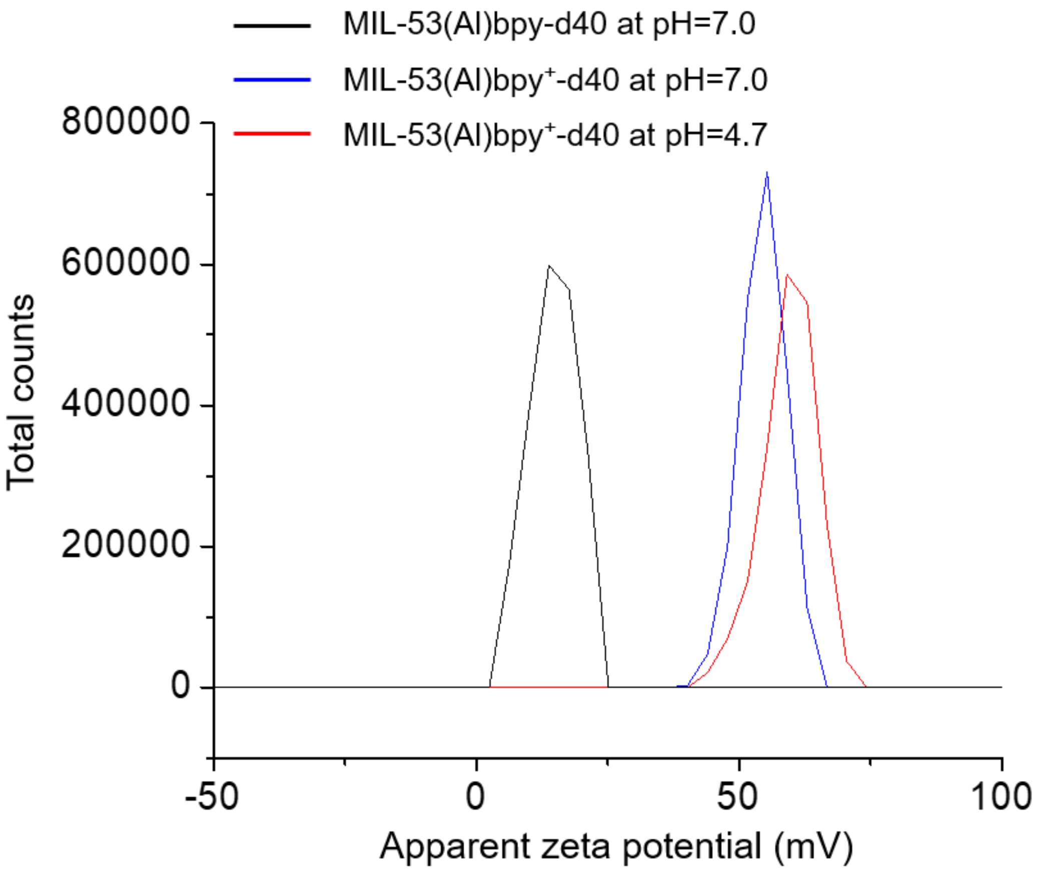

Supplementary Figure 18. Zeta potential of MIL-53(Al)bpy particles at pH 7.0 and MIL-53(Al)bpy^{+}-d40 particles at different pH 4.7 and 7.0 from the MIL-53(Al)bpy^{+}-d40 membrane.

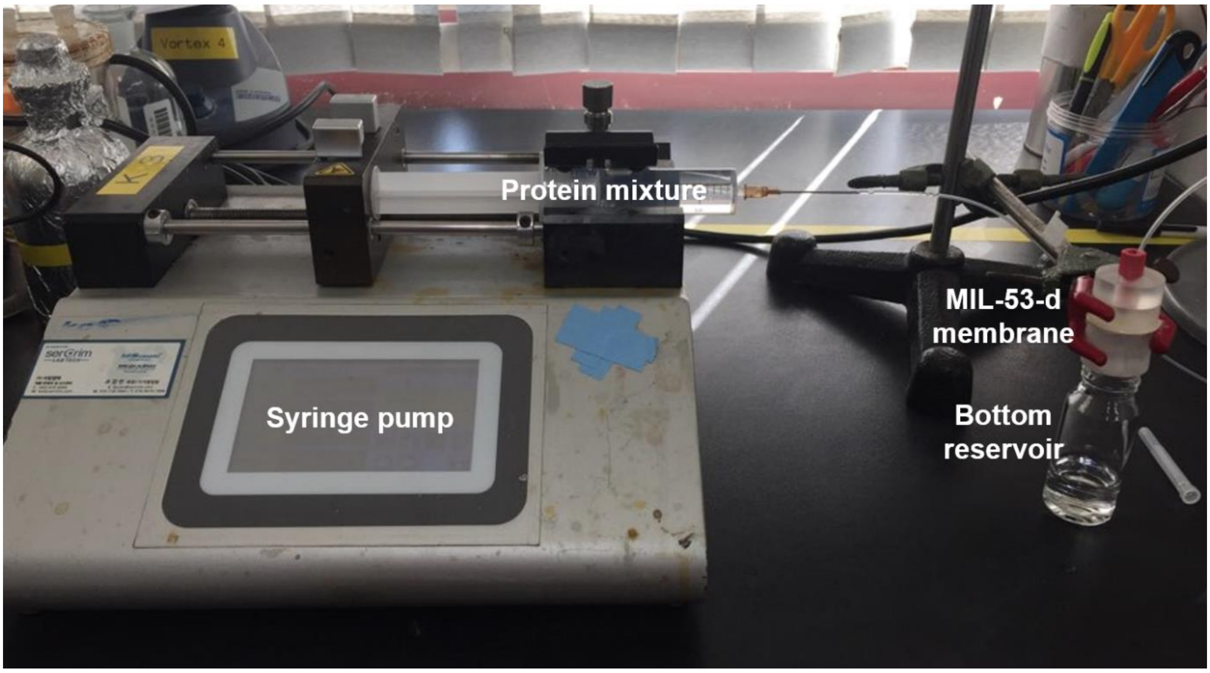

Supplementary Figure 19. General experimental setup for protein separation by MIL-53(Al)bpy^{+}-d40 membrane loaded nanofilter device.

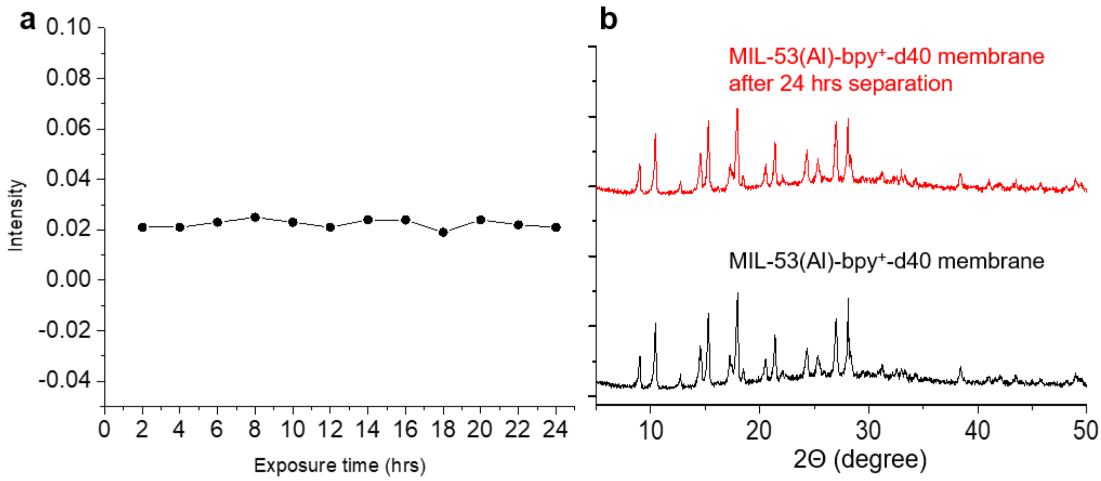

Supplementary Figure 20. (a) Graphical diagram of UV-Vis spectra in real-time monitoring at 280 nm absorbance intensity of BSA collected at bottom reservoir for 24 hrs with 2 hrs interval, (b) X-ray diffraction patterns before and after 24 hrs protein separation.

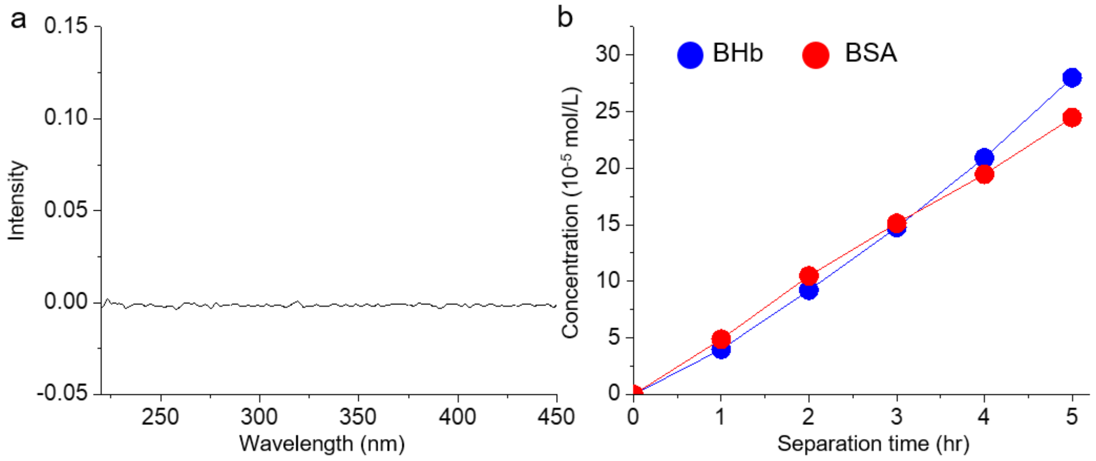

Supplementary Figure 21. (a) Graphical diagram of UV-Vis spectra from 220 to 450 nm wavelength after protein separation experiments with non-decarboxylated MIL-53(Al)bpy^{+} membrane, (b) concentration profiles of the filtered proteins by non-N-quaternized MIL-53(Al)bpy-d40 membrane as a function of separation time at pH 7.0, analyzed by UV-Vis spectra.

Supplementary References

1. Rowsell, J.L.C. & Yaghi, O.M. Effects of Functionalization, Catenation, and Variation of the Metal Oxide and Organic Linking Units on the Low-Pressure Hydrogen Adsorption Properties of Metal−Organic Frameworks. *Journal of the American Chemical Society* **128**, 1304-1315 (2006).
2. Horcajada, P. et al. Synthesis and catalytic properties of MIL-100(Fe), an iron(iii) carboxylate with large pores. *Chemical Communications*, 2820-2822 (2007).
3. Cho, W., Lee, H.J., Choi, S., Kim, Y. & Oh, M. Highly effective heterogeneous chemosensors of luminescent silica@coordination polymer core-shell micro-structures for metal ion sensing. *Scientific Reports* **4**, 6518 (2014).
4. Choi, K.M. et al. Supercapacitors of Nanocrystalline Metal–Organic Frameworks. *ACS Nano* **8**, 7451-7457 (2014).
5. Patil, D.V. et al. MIL-53(Al): An Efficient Adsorbent for the Removal of Nitrobenzene from Aqueous Solutions. *Industrial & Engineering Chemistry Research* **50**, 10516-10524 (2011).
6. Férey, G. et al. A Chromium Terephthalate-Based Solid with Unusually Large Pore Volumes and Surface Area. *Science* **309**, 2040-2042 (2005).
7. Lee, H., Dellatore, S.M., Miller, W.M. & Messersmith, P.B. Mussel-Inspired Surface Chemistry for Multifunctional Coatings. *Science* **318**, 426-430 (2007).
8. Kim, Y. et al. Hydrolytic Transformation of Microporous Metal–Organic Frameworks to Hierarchical Micro- and Meso-porous MOFs. *Angewandte Chemie International Edition* **54**, 13273-13278 (2015).
9. Koo, J. et al. Hollowing out MOFs: hierarchical micro- and meso-porous MOFs with tailorable porosity via selective acid etching. *Chemical Science* **8**, 6799-6803 (2017).
10. Huang, H. et al. An in situ self-assembly template strategy for the preparation of hierarchical-pore metal-organic frameworks. *Nature Communications* **6**, 8847 (2015).
